# Pregnancy and Preeclampsia Affect Monocyte Subsets in Humans and Rats

**DOI:** 10.1371/journal.pone.0045229

**Published:** 2012-09-13

**Authors:** Barbro N. Melgert, Floor Spaans, Theo Borghuis, Pieter A. Klok, Bart Groen, Annemarie Bolt, Paul de Vos, Maria G. van Pampus, Tsz Y. Wong, Harry van Goor, Winston W. Bakker, Marijke M. Faas

**Affiliations:** 1 Division of Medical Biology, Department of Pathology and Medical Biology, University Medical Center Groningen and University of Groningen, Groningen, The Netherlands; 2 Department of Pathology and Medical Biology, University Medical Center Groningen and University of Groningen, Groningen, The Netherlands; 3 Department of Obstetrics and Gynaecology, University Medical Center Groningen and University of Groningen, Groningen, The Netherlands; 4 Department of Pharmacokinetics, Toxicology and Targeting, University of Groningen, Groningen, The Netherlands; Otto-von-Guericke University Magdeburg, Germany

## Abstract

**Introduction:**

Both nonclassical and intermediate monocytes have been implicated in different inflammatory conditions. We hypothesized that these monocytes would increase during pregnancy, a condition associated with generalized activation of inflammatory responses and that they would increase even more during preeclampsia, in which inflammatory responses are further stimulated. In the present study we investigated changes in monocyte subsets during healthy pregnancy and preeclampsia in humans and rats.

**Methods:**

Blood monocyte subsets of nonpregnant, preeclamptic and healthy pregnant women were identified with CD14 and CD16. In nonpregnant and pregnant rats, blood monocytes were identified with CD172a and CD43, as well as in rats infused with adenosine triphosphate (ATP), a pro-inflammatory stimulus known to induce preeclampsia-like symptoms. Total and CD206-positive macrophages were quantified in placentas of these animals.

**Results:**

Lower percentages of classical monocytes were found in pregnant women (91%–[83–98%]) compared to nonpregnant women (94%–[90–98%]) and even less in preeclamptic patients (90%–[61–92%]). In contrast, the percentage of combined nonclassical/intermediate monocytes was higher in pregnant women (8.5%–[2.3–16.6%] vs. 5.6%–[1.9–9.5%]) and even higher in preeclamptic patients (9.9%–[7.8–38.7%]), which was caused by a selective increase of intermediate monocytes. In rats, we also found lower percentages of classical monocytes and higher percentages of nonclassical monocytes in pregnant versus nonpregnant rats. ATP infusion increased the percentage of nonclassical monocytes in pregnant rats even further but not in nonpregnant rats. These nonclassical monocytes showed a more activated phenotype in pregnant ATP-infused rats only. Mesometrial triangles of ATP-infused rats had less CD206-positive macrophages as compared to those of saline-infused rats.

**Conclusion:**

The higher percentage of nonclassical/intermediate monocytes found in pregnancy and preeclampsia confirms their association with inflammatory responses. The observation that ATP stimulated numbers/activation of nonclassical monocytes in pregnant rats *only*, suggests that nonclassical monocytes are specifically altered in pregnancy and may play a role in the pathophysiology of preeclampsia.

## Introduction

During pregnancy the maternal immune system adapts to accommodate the semi-allogeneic fetus [1], [2]. A major alteration involves significant activation of the innate immune system as reflected by an increase in the number of peripheral blood monocytes [3]–[7]. From the second trimester onwards these cells also display an activated phenotype comparable to that seen in systemic sepsis [7]–[11]. Human monocytes consist of 3 subsets according to the Nomenclature Committee of the International Union of Immunological Societies [12] and appear to have different functions [13]. Whether the changes in monocyte number and behavior during pregnancy are the result of changes in monocyte subsets is unknown and is the subject of the present communication.

The new classification of monocytes is based on the expression of CD14 (receptor for lipopolysaccharide) and CD16 (Fcγ receptor III) [12]. CD14-hi CD16-neg monocytes are commonly referred to as the “classical subset”. Classical monocytes are associated with extravasation and inflammation. Once infiltrated into tissues, these monocytes develop into macrophages and help with pathogen clearance and wound healing [14]. CD14-lo CD16-hi monocytes are commonly referred to as the “nonclassical subset” and CD14-hi CD16-int monocytes as the “intermediate subset”. The intermediate subset has been suggested to be a transitional population between the classical and the nonclassical subsets [12] and has often been grouped together with the nonclassical subset in literature, collectively also called the nonclassical subset. The function of the intermediate subset is not known, but the combined nonclassical and intermediate subsets have been thought to replenish tissue resident macrophages and dendritic cells [14] and have been shown to produce pro-inflammatory cytokines [12], [15]. Their pro-inflammatory association was also illustrated by the fact that their number increases during inflammatory conditions, such as during sepsis [12], [16], [17].

In rats, a similar subdivision of monocyte subsets as found in humans can be made based on the marker CD43 [18]. A CD43-lo subset was found to be similar to the classical subset and a CD43-hi subset was found to be similar to the nonclassical subset in humans [14]. The rat could thus potentially serve as a model for studying monocyte subsets during pregnancy.

In the present study we investigated monocyte subsets in peripheral blood of pregnant and nonpregnant women and rats. We hypothesized that as pregnancy is considered a pro-inflammatory condition, in particular nonclassical/intermediate monocytes would be increased in this condition. In addition to healthy pregnancy, we also investigated monocyte subsets in subjects with preeclampsia, a major pregnancy complication with unknown etiology and only partly known pathogenesis [19]. This disorder originates in the placenta, which contains ischemic areas. Circulating factors in maternal blood, produced by the ischemic placenta, cause endothelial dysfunction and systemic inflammatory responses leading to the clinical manifestations of the disease, i.e. hypertension, proteinuria and edema [20]. The systemic inflammatory response is reflected by a significant increase in peripheral blood monocytes and an altered activation status of these monocytes as compared to healthy pregnancy [21]–[26]. We therefore hypothesized that during preeclampsia nonclassical/intermediate monocytes may even be further increased as compared to normal pregnancy.

Changes in monocytes during preeclampsia could result from the presence of pro-inflammatory factors. One of the pro-inflammatory factors found elevated in blood of preeclamptic women is adenosine triphosphate (ATP) [28]. We have previously shown that ATP infusion in pregnant rats is pro-inflammatory and induces preeclampsia-like symptoms [29]. Therefore, ATP could be a factor affecting monocyte subsets in pregnant individuals. We thus also studied whether infusing pregnant rats with the pro-inflammatory stimulus ATP could induce changes in monocyte subsets similar to those seen in human preeclampsia.

Monocytes were shown to infiltrate the decidua with the onset of pregnancy and develop into regulatory macrophages and immature dendritic cells [30]–[34].These macrophages have been postulated to be essential for proper spiral artery remodeling and inducing tolerance of the fetus [32], [35], [36], but it is unknown if they develop from one specific subset of monocytes. Therefore, we also studied placentas of these pregnant rats to evaluate whether ATP would affect the number of regulatory macrophages in the decidua.

## Materials and Methods

### Ethics statement

The present human study was performed after approval of the Medical Ethics Committee (application number NL25930.042.08) at the University Medical Center Groningen. Signed informed consent forms were received from all participants.

All animal experiments were approved by the Institutional Animal Care and Use Committee of the University of Groningen (application number DEC-4270C). All surgeries were performed under isoflurane/oxygen anesthesia and all efforts were made to minimize suffering including the use of buprenorphine for pain management after surgery.

### Patients

Patients with early-onset preeclampsia (n = 26) and healthy pregnant controls (n = 23) were recruited from the antenatal ward of the University Medical Center Groningen. Preeclampsia was defined according to the standards of the International Society for the Study of Hypertension in Pregnancy (ISSHP): diastolic blood pressure of 90 mmHg or more on two or more consecutive occasions more than 4 hrs apart and proteinuria of more than 300 mg/24 hours [37]. We included early-onset preeclamptic women, defined as the onset of preeclampsia before 34 weeks. Blood samples were taken from nonpregnant women and from patients with preeclampsia and healthy pregnant women between week 28 and 32 of pregnancy. The nonpregnant women (n = 19) were recruited from hospital staff and students. Exclusion criteria for all groups were pre-existing hypertension, diabetes mellitus, vasculitis, renal disease, autoimmune disease, malignancies or women who had recent trauma or surgery.

Maternal blood samples of both pregnant and preeclamptic women were collected during routine blood sampling. Blood samples were drawn from the antecubital vein into 10 mL tubes containing EDTA (Venoject, Terumo Europe NV, Leuven, Belgium). Samples were immediately prepared for flow cytometry. See Table 1 for patient characteristics.

**Table 1:** Patient characteristics of nonpregnant, healthy pregnant and preeclamptic women.

	*Nonpregnant (n = 19)*	*Pregnant (n = 23)*	*Preeclampsia (n = 26)*
**Maternal age (Y)**	26.5 (23–35)	30 (21–39)	30 (23–40)
**Caucasian**	19	23	26
**Blood sampling**			
Gestational age (wk)	NA	29.8 (28–32)	30 (23–31.0)
Systolic blood pressure (mmHg)	nd	nd*	160 (140–200)
Diastolic blood pressure (mmHg)	nd	nd*	105 (90–115)
Proteinuria (g/24 hr)	nd	nd*	1.1 (0.3–7.1)
**Delivery**			
Gestational age (wk)	NA	40 (38.4–42)	30.6 (25.8–34.3)#
Birth weight (g)	NA	3570 (2820–4430)	1123.5 (530–2000)#

Medians plus range are shown. Mann Whitney U comparisons were made between nonpregnant – pregnant women and pregnant – preeclamptic women, significant differences (p<0.01) are indicated by #.

nd: not determined; NA: not applicable.

* : Blood pressure and protein content of urine of healthy pregnant women was within normal ranges and not routinely recorded.

### Human blood leukocyte counts

Before cell staining for flow cytometry, part (500 µl) of human blood samples was used to establish total blood leukocyte numbers. Leukocytes were counted using a microcell counter (model Sysmex PocH 100i, Sysmex Netherlands, Etten-Leur, the Netherlands).

### Staining of human blood for flow cytometry

All blood samples were immediately diluted with RPMI-1640 (1∶1) and incubated with PE-conjugated anti-CD16 antibodies (Biolegend, Uithoorn, the Netherlands) and with FITC-conjugated anti-CD14 antibodies (Biolegend) for 30 minutes in the dark. Subsequently the red blood cells were lysed using BD FACS^TM^ Lysing Solution (BD Biosciences, Breda, the Netherlands) for 5 minutes. The cells were then washed twice (with 1% bovine serum albumin in PBS), fixed with 2% paraformaldehyde and kept in the dark at 4°C (no longer than one day) until FACS analysis. All steps were performed at room temperature.

### Data collection and evaluation

Data were collected on a BD LSR II Flow Cytometer (BD Biosciences) and were analyzed using FlowJo software (Tree star, Inc., Ashland, OR, USA). At least 200.000 events were counted per analysis.

**Figure 1:**
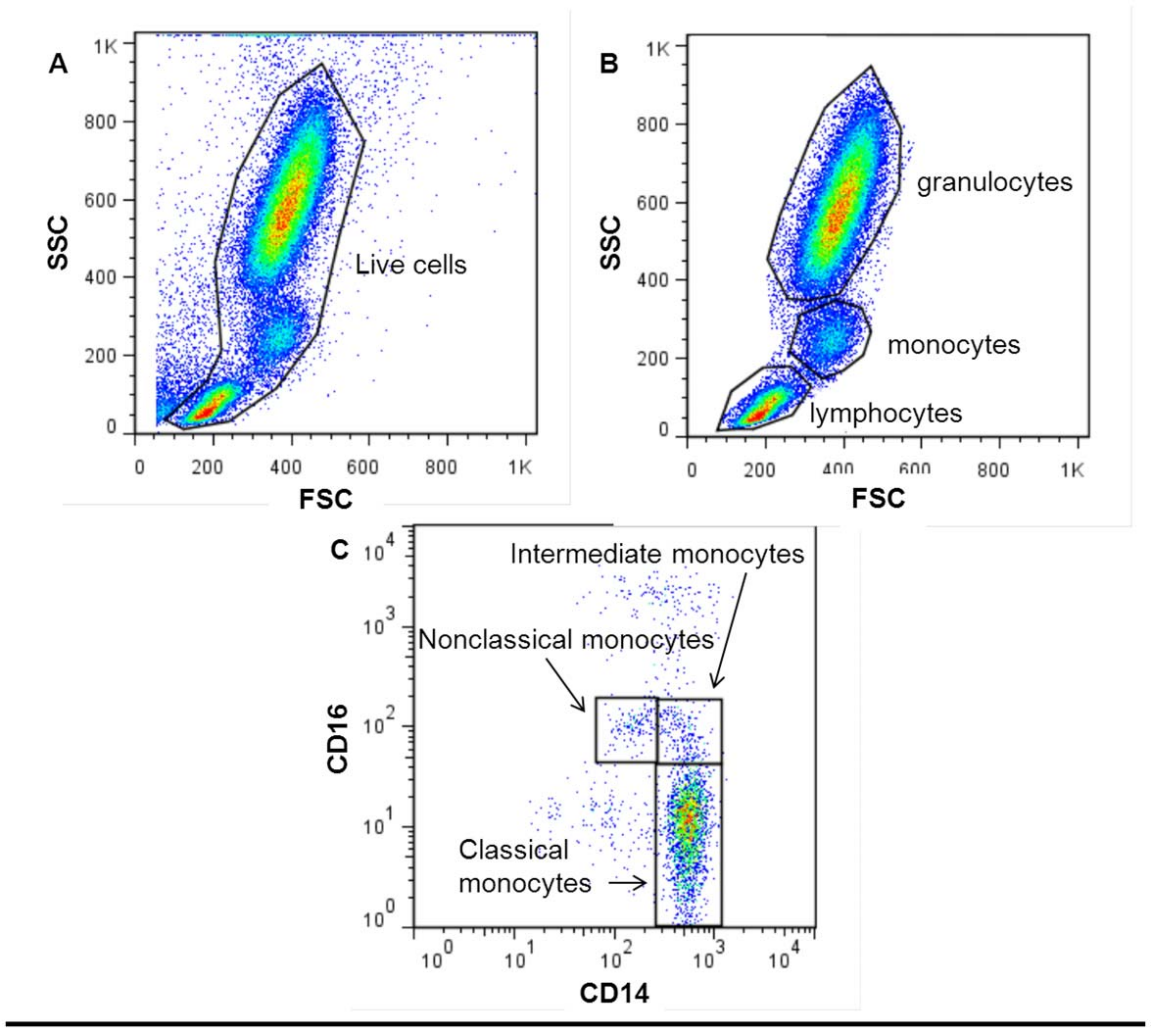
Flow cytometric gating strategy for monocyte subsets in human peripheral blood. Dead cells were excluded by selecting all live cells from the forward/side scatter plot (A), after which monocytes were selected (B). Subsequently, cells were plotted for their CD14 and CD16 expression, after which three distinct subpopulations of monocytes (classical, nonclassical and intermediate) could be selected and analyzed (C).

In the gating strategy used for analysis of the three monocyte subsets in human blood, live cells were first selected from the forward/side scatter plot (see Figure 1A). Subsequently the monocytes were selected from the live cells in the forward/side scatter plot and were then copied to a CD14/CD16 plot (see Figure 1B). Three subsets were identified: CD14-hi CD16-neg (classical subset), CD14-lo CD16-hi (nonclassical subset) and CD14-hi CD16-int (intermediate subset) (see Figure 1C). The percentages of the three populations within the monocyte population were calculated (% of monocytes). We used the official nomenclature for the 3 subsets, but for the sake of comparing with other, older publications that did not make a distinction between nonclassical and intermediate monocytes, we also used the combined nonclassical/intermediate subset. Absolute monocyte numbers were calculated using the leukocyte count and percentage of monocytes in the population of live cells.

### Animals

Female Wistar outbred rats (about 200 g) were kept in a temperature and light-controlled room (lights on from 7∶30 AM until 7∶30 PM) with free access to food and water. Until selection for experiments, vaginal smears were taken daily, and rats were rendered pregnant by housing them on pro-oestrus with fertile males for one night.

When spermatozoa were detected in the smear the next day, this day was designated as day 0 of pregnancy. In a number of the rats, a cannula was inserted into the right jugular vein under isoflurane/oxygen anesthesia as described previously [38]. This cannula allows stress-free blood sampling and infusions. All efforts were made to minimize suffering of animals.

### Experimental design animal experiment

In an initial experiment we tested whether in rat pregnancy, similar to human pregnancy, monocyte subsets changed. In this experiment, pregnant (day 20; n = 5) and nonpregnant (n = 5) rats were sacrificed by aortic punction under anesthesia (isoflurane/oxygen). EDTA blood samples were taken for analysis of leukocyte counts and flow cytometry.

In a second experiment, rats were infused with the pro-inflammatory stimulus ATP [29], [39] in order to evaluate whether a pro-inflammatory stimulus during pregnancy increases the nonclassical monocyte subset. Pregnant rats were infused with 3000 μg/kg bw ATP in 2.0 ml saline (n = 9) or with 2.0 ml saline alone (n = 7) on day 14 of pregnancy as described before [29]. Nonpregnant rats were infused with 3000 μg/kg bw ATP in 2.0 ml saline (n = 6) or saline alone (n = 5) on di-oestrus. For determination of leukocyte counts and for flow cytometry EDTA blood samples (0.4 ml) were taken from the jugular vein cannula 7 days before (day 7), 1 day before (day 13), 1 day after (day 15) and 3 days after (day 17) the infusion. Blood samples on day 20 were taken during sacrifice by aortic punction under anesthesia (isoflurane/oxygen). At the time of sacrifice, placentas with mesometrial triangle were dissected from the uterine horn and fixed in paraformaldehyde (4%) for 24 hrs. They were then transferred to alcohol (70%) until embedding in paraffin.

### Rat blood leukocyte counts

Before cell staining for flow cytometry, 20 µl of EDTA rat blood was diluted in 500 µl pOCH buffer (Sysmex Netherlands) for leukocyte counts. Leukocytes were counted using a microcell counter (model Sysmex PocH 100i, Sysmex Netherlands).

### Staining of rat blood for flow cytometry

Whole blood samples were stained to identify monocyte subsets (i.e. for CD172a and CD43 expression) and in rats from experiment 2 the activation status of monocytes was also investigated using CD4 and CD62L expression. Samples were washed with PBS and subsequently incubated with PE-conjugated anti-CD172a (Biolegend) and AlexaFluor647-conjugated anti-CD43 (Biolegend) to identify the two monocyte subsets (30 minutes in the dark). In addition, samples of rats from experiment 2 were also incubated with FITC-conjugated anti-CD4 (BD Pharmingen) and PercP-conjugated anti-CD62L (Biolegend), or with isotype controls to assess the activation status of monocytes. After antibody incubation, cells were washed with 0,5 ml FACS buffer (PBS with 2% fetal calf serum and 1,3 mM EDTA) and red blood cells were lysed and leukocytes fixed by incubation with BD FACS^TM^ Lysing Solution (BD Biosciences) for 30 minutes. Cells were subsequently washed twice, resuspended in FACS buffer and kept in the dark at 4°C (no longer than one day) until FACS analysis. All steps were performed at room temperature.

### Data collection and evaluation

Data were collected on a BD LSR II Flow Cytometer (BD Biosciences) and were analyzed using FlowJo software (Tree star). At least 200.000 cells were counted per analysis.

**Figure 2:**
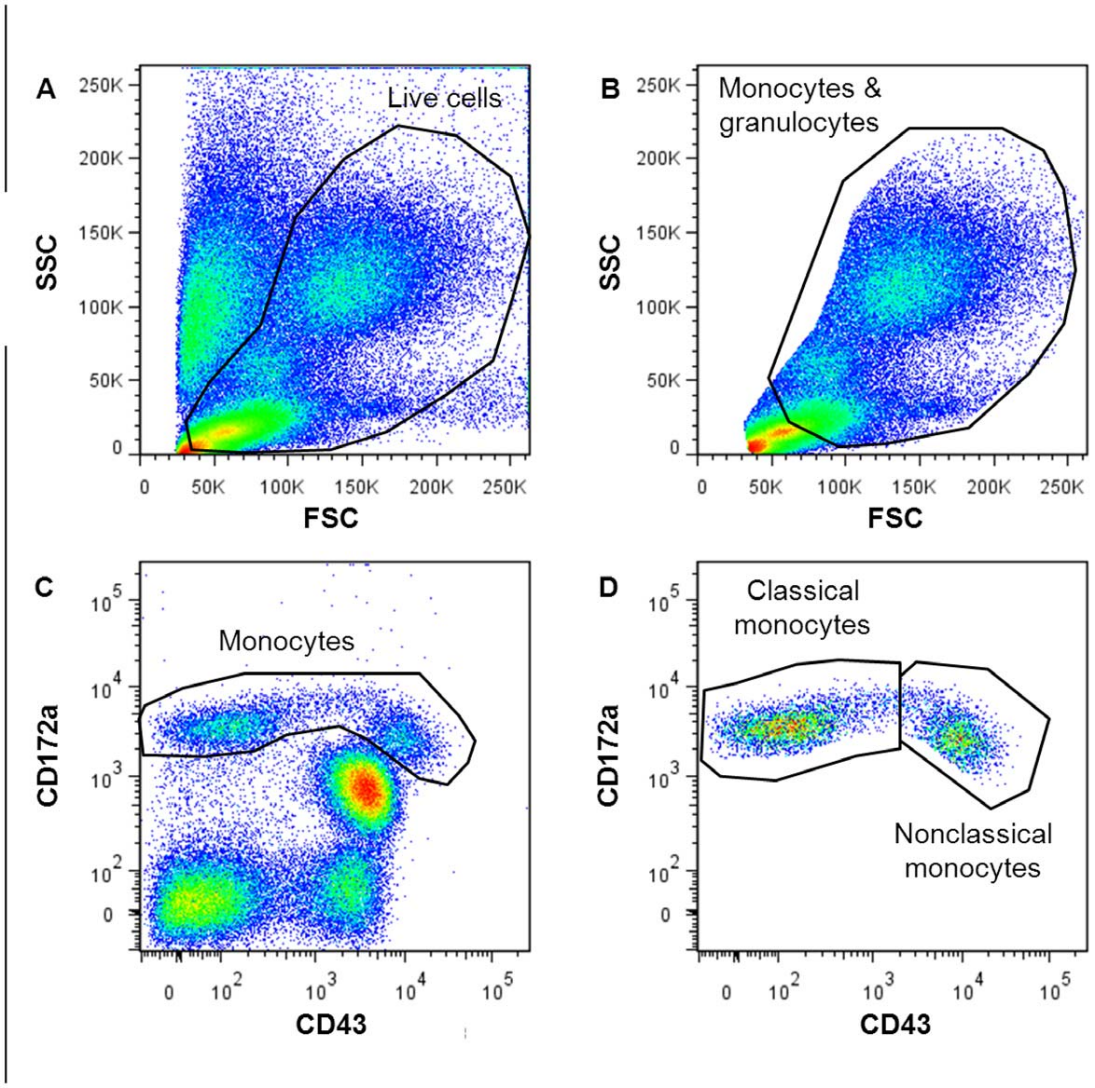
Flow cytometric gating strategy for monocyte subsets in rat peripheral blood. Dead cells and red blood cells were excluded by selecting all live cells from the forward/side scatter plot (A), after which a wide gate around the monocytes and granulocytes was selected (B) and copied to a CD172a/CD43 plot (C). The total monocyte population (used for calculating the total monocyte numbers) was selected from the CD172a/CD43 plot (C) and subsequently plotted in a new graph to calculate the percentages of the two classical (CD43-lo) and nonclassical (CD43-hi) subsets in the total monocyte population (D).

In the gating strategy used for analysis of monocyte subsets in rat blood, live cells were first selected from the forward/side scatter plot (see Figure 2A). From the live cells a wide gate around the monocytes was drawn (rat monocytes are often not a distinct population in the forward/side scatter, thus to be sure to include all monocytes we decided to set a wider gate in the forward/side scatter) and copied to a dot plot in which the classical (CD43-lo) and nonclassical (CD43-hi) monocytes were identified based on expression of CD43 and CD172a (see Figure 2B–D).

To analyze CD4 and CD62L expression on monocyte subsets, blood samples stained with isotype controls were used to set a gate that for each fluorescent label (FITC for CD4 and PercP for CD62L) contained no more than 1% of the population. This gate was subsequently copied to the CD4 and CD62L stained samples, after which the mean fluorescent intensity (MFI) of CD4 and CD62L expression on monocytes was calculated.

From these data either the percentage of monocytes of a certain subset within the total monocyte gate (% of monocytes) or the percentage of a monocyte subset in the whole live population (% of live) was calculated. Per rat percentage deviation from day 13 was calculated. Absolute monocyte numbers were calculated using the leukocyte count and percentage of CD172a+ cells of the population of live cells.

### CD68 and CD206 immunohistochemistry

Total macrophages/monocytes and M2-like macrophages were identified by staining for CD68 (1∶100 diluted, clone ED1, AbD Serotec, Düsseldorf, Germany) and CD206 (1∶1000 diluted; Abcam, Cambridge, UK) respectively according to standard methods. In short, paraformaldehyde (4%) fixed paraffin sections (4 µm) of placentae from rats infused with saline or ATP were deparaffinized and incubated in 10 mM Tris/1 mM EDTA buffer pH = 9 (for CD68, 15 min.) or in 10 mM citrate buffer pH = 6 (for CD206, 10 min.) at 100°C for antigen retrieval. After incubation with 10% normal goat serum (30 min.) mouse-anti-CD68 or rabbit-anti-CD206 antibodies were used as a first step (60 min.). Sections were washed with PBS (pH = 7.2) and endogenous peroxidase activity was blocked with 3% H_2_O_2_ in methanol and endogenous avidin/biotin activity was blocked with a Biotin blocking kit (Dako, Heverlee, Belgium). After washing with PBS, biotin-conjugated goat-anti-mouse (for CD68, Southern Biotech, Birmingham, AL, USA) and biotin-conjugated goat-anti-rabbit (for CD206, Dako) were added as a second step (30 min.). Sections were washed with PBS, peroxidase conjugated streptavidin (Dako) was added (30 min.) and the staining was subsequently visualized by 3-amino-9-ehtyl-carbazole. Haematoxylin was used as a nuclear staining. All of the incubation steps of the staining procedures were carried out at room temperature. Control sections, which were consistently negative, were sections incubated without the first antibody.

### Morphometric analysis of immunohistochemical stainings

Sections stained for CD68 and CD206 were scanned using an Aperio ScanScope CS and analyzed with Aperio ImageScope v10.2.2.2319 (Aperio, Vista, CA, USA). The ‘Positive pixel Count V9’ algorithm was used to analyze CD68 or CD206-positive (red) pixels as well as total unstained tissue pixels in both the decidua and the mesometrial triangle (comparable to human placental bed) of the rat placenta. Subsequently these data were used to calculate the percentage of CD68 or CD206-positive tissue in each of the two compartments.

### Statistical analysis

For statistical analysis of differences in monocyte subsets in human blood samples we used a nonparametric Kruskall Wallis test followed by Mann Whitney U tests. For testing differences between monocyte subsets in the first rat experiment and testing differences in staining for CD68 and CD206 Mann Whitney U tests were performed as well. For statistical analysis of the longitudinal data in the second rat experiment nonparametric Friedman repeated measures tests were performed followed by Dunn's posttests comparing day-13 pre-infusion values with day 15, 17 or 20 post-infusion values. Differences were considered to be significant if p<0.05 and a statistical trend if p<0.1. The data are presented as medians with either the range or the interquartile range depicted.

## Results

### Human peripheral blood monocyte subsets in pregnant and preeclamptic individuals

Compared to nonpregnant women, a significantly higher number of leukocytes was found in healthy pregnant women and an even higher number in preeclamptic women as compared to healthy pregnant women (Table 2). Also, total monocyte numbers were significantly higher in healthy pregnant women compared to nonpregnant women (Table 2).

**Table 2:** Leukocyte and monocyte counts in the human peripheral blood samples of nonpregnant, healthy pregnant and preeclamptic women.

	*Nonpregnant (n = 19)*	*Pregnant (n = 23)*	*Preeclampsia (n = 26)*
**Leukocyte count** (×10^9^ cells/L)	6.5 (4.2–11.2)	8.5 (5.8–14.2)#	10.5 (6.1–18.2)*
**Total monocyte** **count** (×10^9^ cells/L)**	0.3 (0.1–0.8)	0.6 (0.4–0.9)∧	0.5 (0.1–1.1)

Medians plus range are shown. Mann Whitney U comparisons were made between nonpregnant – pregnant women and pregnant – preeclamptic women. #: p<0.01 pregnant compared to nonpregnant women, *: p<0.05 preeclamptic compared to healthy pregnant women, ∧: p<0.0001 pregnant compared to nonpregnant women.

** : as determined from the leukocyte counts and percentage of monocytes in the population of live cells of the flow cytometric analysis.

**Figure 3:**
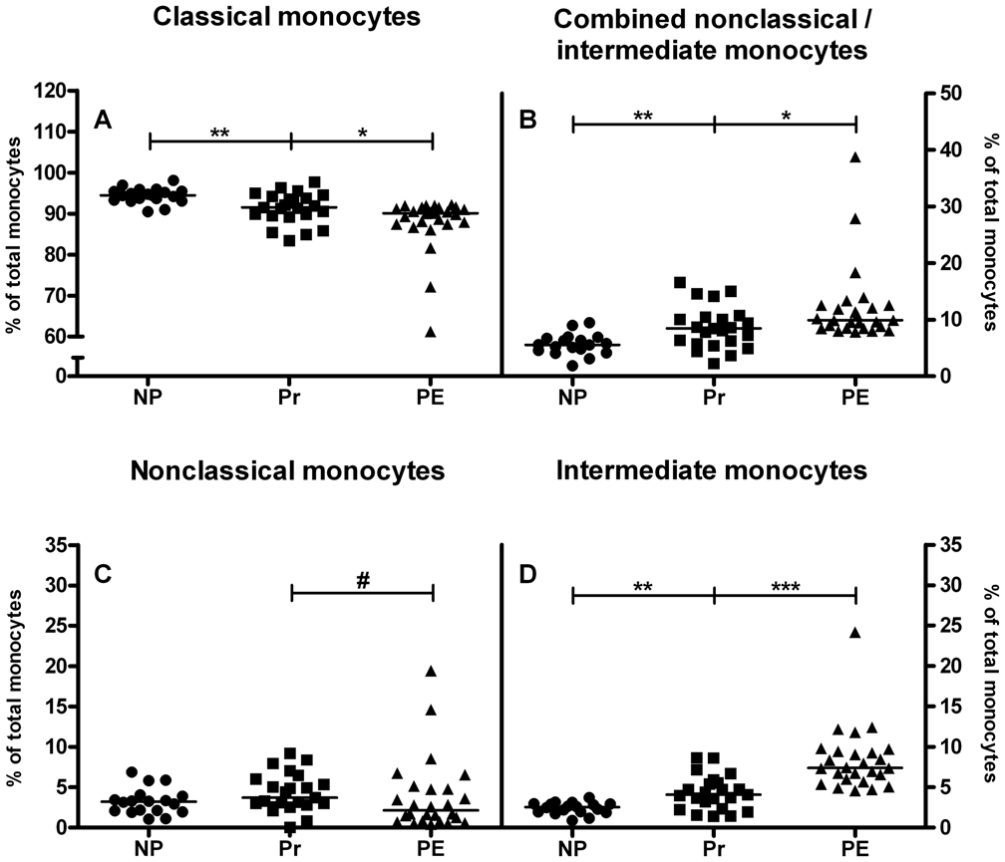
Human monocyte subsets in peripheral blood. Lower percentages of classical monocytes were observed in healthy pregnant women (Pr, squares) as compared to nonpregnant women (NP, circles) and even less in preeclamptic women (PE, triangles) compared to healthy pregnant women (A). Higher percentages of combined nonclassical/intermediate monocytes were found in pregnant women compared to nonpregnant women and even higher percentages in preeclamptic patients compared to healthy pregnant women (B). When subdividing into nonclassical and intermediate monocytes, no significant changes were observed in nonclassical monocytes (C), though a trend towards lower nonclassical monocytes was observed in preeclamptic women compared to healthy pregnant women. In contrast, higher percentages of intermediate monocytes were found in healthy pregnant women compared to nonpregnant women and even higher percentages in preeclamptic patients compared to healthy pregnant women (D). Medians are shown, #p<0.1, *p<0.05; **p<0.01; ***p<0.001; Mann Whitney U test.

To study the changes in monocyte subsets during pregnancy and preeclampsia, peripheral blood of nonpregnant, healthy pregnant and preeclamptic women was stained for the 3 subsets, i.e. the classical, the nonclassical and the intermediate subsets. Significantly lower percentages of classical monocytes (defined as the percentage of the total monocyte population) were found in healthy pregnant women as compared to nonpregnant controls (p<0.01, Figure 3A). In preeclamptic patients, the percentage of classical monocytes was even lower as compared to healthy pregnant women (p<0.05, Figure 3A). The percentage of the combined nonclassical/intermediate monocytes, on the other hand, was significantly higher in healthy pregnant women compared to nonpregnant women (p<0.01, Figure 3B) and even higher in preeclamptic patients as compared to healthy pregnant women (p<0.05, Figure 3B).

Division of the combined nonclassical/intermediate monocytes into the nonclassical subset and the intermediate subset [12] showed no significant differences in the percentage of nonclassical monocytes between the three groups (Figure 3C), though a trend towards lower nonclassical monocytes was observed in preeclamptic women compared to healthy pregnant women. In contrast, significantly higher percentages of intermediate monocytes were found in peripheral blood of healthy pregnant women as compared to nonpregnant women (p<0.01, Figure 3D) and even higher percentages in peripheral blood of preeclamptic patients as compared to healthy pregnant women (p<0.001, Figure 3D).

### Rat peripheral blood monocyte subsets during pregnancy

**Figure 4:**
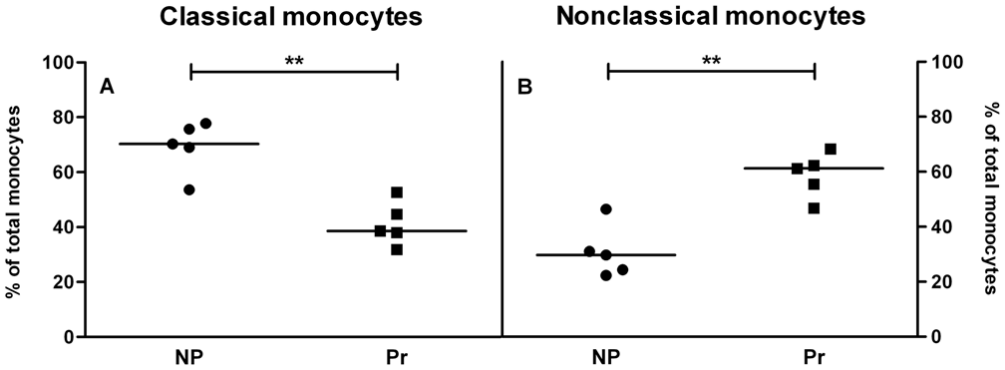
Rat monocyte subsets in peripheral blood on day 20 of pregnancy. At the end of pregnancy (day 20) in rats (Pr, squares), the percentage of classical monocytes decreases (A) and the percentage of nonclassical monocytes rises (B) as compared to nonpregnant rats (NP, circles). Medians are shown, **p<0.01; Mann Whitney U test.

Both peripheral blood leukocyte numbers (trend) and total monocyte numbers (p<0.05) were higher in pregnant rats compared to nonpregnant rats (Table 3). In rats, only two monocyte subsets are distinguished, the nonclassical (CD43-hi) and the classical (CD43-lo) monocytes. To evaluate whether similar changes in monocyte subsets in rat pregnancy occur as in human pregnancy, we compared the percentages of classical and nonclassical monocytes in peripheral blood between nonpregnant and pregnant rats at the end of pregnancy (day 20). In pregnant rats, the percentage of classical monocytes was significantly lower while the percentage of nonclassical monocytes was significantly higher compared to nonpregnant rats (p<0.01, Figure 4A and B).

**Table 3:** Leukocyte and monocyte counts in peripheral blood samples of nonpregnant and pregnant rats.

	*Nonpregnant (n = 5)*	*Pregnant (n = 5)*
**Leukocyte count** (×10^9^ cells/L)	3.2 (2.1–5.1)	5.6 (3.7–7.0)#
**Total monocyte** **count** (×10^9^ cells/L)**	0.13 (0.07–0.49)	0.57 (0.29–0.59)*

Medians plus range are shown. Mann Whitney U comparisons were made between nonpregnant and pregnant rats, significant differences (p<0.05) are indicated by *, a statistical trend (p<0.1) by #.

** : as determined from the leukocyte counts and percentage of CD172a+ cells of the population of live cells of the flow cytometric analysis.

### Effect of pro-inflammatory ATP on monocyte subsets during rat pregnancy

As pregnancy and preeclampsia are pro-inflammatory conditions, we investigated in a second experiment whether a pro-inflammatory stimulus during pregnancy was able to induce the increase in nonclassical monocytes seen in human pregnancy and preeclampsia. As pro-inflammatory stimulus we used ATP, because it is increased in preeclampsia [28] and induced preeclamptic-like symptoms in pregnant rats [29].

**Figure 5:**
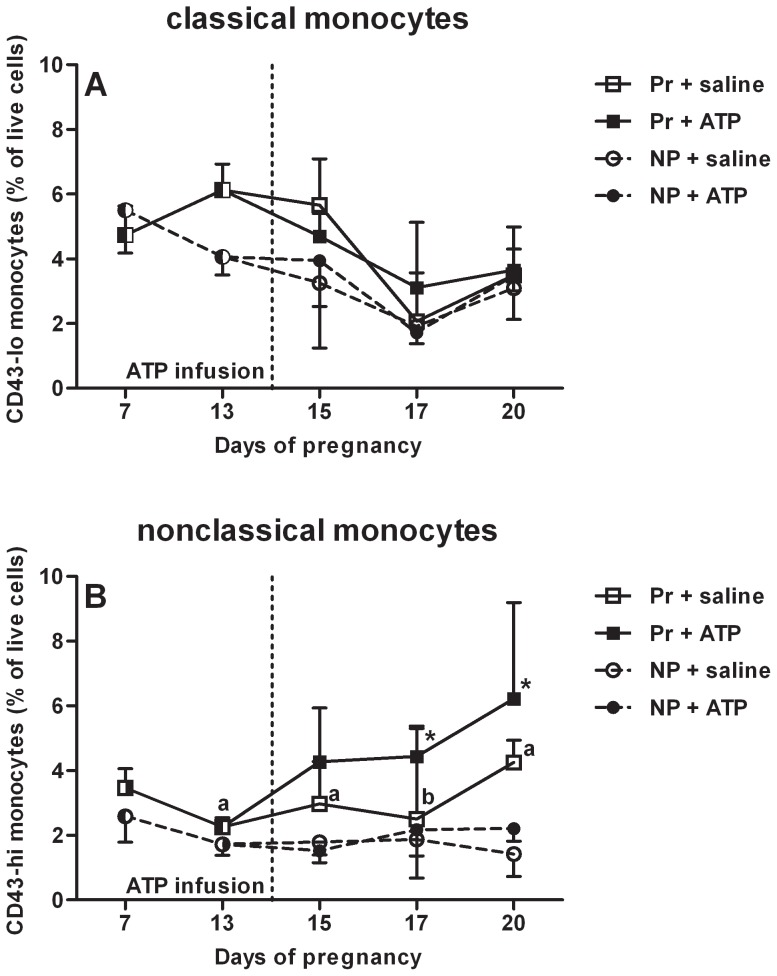
Rat monocyte subsets after ATP or saline infusion. No changes in classical monocytes (as percentage of live cells) (A) were found after ATP (solid line, solid symbols) or saline infusion (dashed line, open symbols) between pregnant (Pr, squares) and nonpregnant (NP, circles) rats infused on day 14 of pregnancy, while the percentage of nonclassical monocytes (B) increased after ATP-infusion in the pregnant rats only. Medians with the interquartile range are shown, *p<0.05 in a Friedman repeated measures test followed by Dunn's posttest compared to day 13 (pre-infusion). a = p<0.05 and b = p<0.1 in pregnant saline vs nonpregnant saline as tested with Mann Whitney U for the same day. NP + saline n = 5; NP + ATP n = 6; Pr + saline n = 7; Pr + ATP n = 9.

As controls we used pregnant saline-infused rats, which allowed us to study the course of changes in monocyte subsets during pregnancy. Figures 5A and B show that CD43-lo monocytes are not significantly different between pregnant and nonpregnant animals during the course of pregnancy (comparisons were made between the saline-treated pregnant and saline-treated nonpregnant rats) and that CD43-hi monocytes are higher in pregnant rats from day 13 onwards.

No effects of ATP infusion as compared with saline infusion were observed on the percentages of monocyte subsets in nonpregnant rats (Figures 5A and 5B). However, in pregnant rats, infusion with ATP induced a significant increase in the percentage of nonclassical monocytes on day 17 and 20 of pregnancy compared to pre-infusion values on day 13, while this significant increase was not observed in the saline-infused pregnant animals (p<0.05, Figure 5B). No changes were found in the percentage of classical monocytes after ATP or saline infusion in pregnant or non-pregnant rats (Figure 5A).

**Figure 6:**
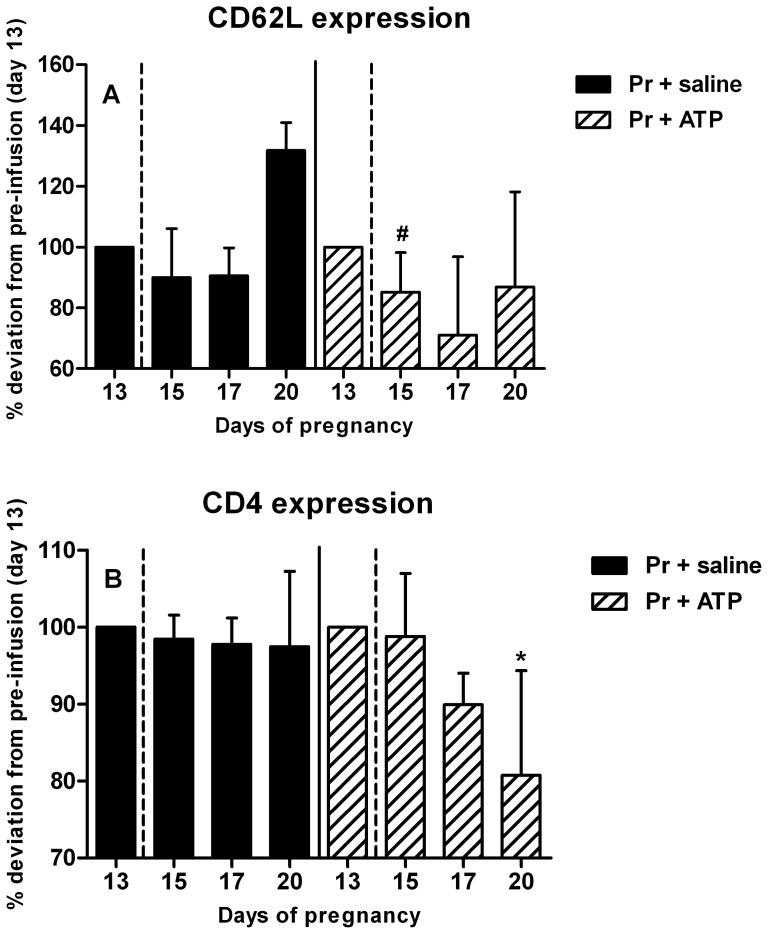
Activation of nonclassical monocytes after ATP infusion in pregnant rats. Activation of nonclassical monocytes was assessed by lower mean fluorescent intensity (MFI) of two activation markers, CD62L (A, early activation marker) and CD4 (B, late activation marker) [21], [40]. Decreased expression of CD62L was found in ATP-infused rats only on nonclassical monocytes on day 15 (trend) of pregnancy compared to pre-infusion day 13, while CD4 expression on nonclassical monocytes was decreased on day 20 of pregnancy compared to pre-infusion day 13 in the same experimental group. Percentage deviations from pre-infusion day 13 are shown. Medians with the interquartile range are shown, #p<0.1 (trend), *p<0.05 in a Friedman repeated measures test followed by Dunn's posttest compared to day 13 (pre-infusion). NP + saline n = 5; NP + ATP n = 6; Pr + saline n = 7; Pr + ATP n = 9.

To investigate whether ATP also activated monocytes, we evaluated CD62L and CD4 expression before and after infusion with ATP or saline in pregnant rats and in nonpregnant rats. CD62L and CD4 are early and late activation markers of rat monocytes, respectively, and their expressions are known to decrease after activation [21], [40]–[43]. One day after ATP infusion in pregnant rats, expression of the early activation marker CD62L on nonclassical monocytes was decreased (trend p<0.1 on day 15, Figure 6A) as compared to pre-infusion values. No differences were found at later time points for this marker. CD4 expression on nonclassical monocytes on the other hand was not significantly different from pre-infusion values shortly after ATP infusion (days 15 and 17), but was significantly decreased on day 20 (p<0.05 on day 20, Figure 6B). No changes in CD4 and CD62L expression were observed on classical monocytes after ATP or saline infusion in pregnant rats (data not shown). Moreover, no effects of CD62L and CD4 expression on either classical or nonclassical monocytes were observed after ATP or saline infusion in nonpregnant rats (data not shown).

### Effect of pro-inflammatory ATP on total and M2-like macrophages in rat placenta

To investigate whether the changes we found in monocyte subsets were associated with changes in M2-like/regulatory macrophages in the placenta, we quantified the percentage total macrophages (CD68-positive macrophages) and M2-like/regulatory macrophages (CD206-positive macrophages) in the decidua and mesometrial triangle (comparable to human placental bed) of placentas of rats exposed to saline or ATP (by quantifying the percentage of positively-stained area in the total decidual/mesometrial triangle area).

**Figure 7:**
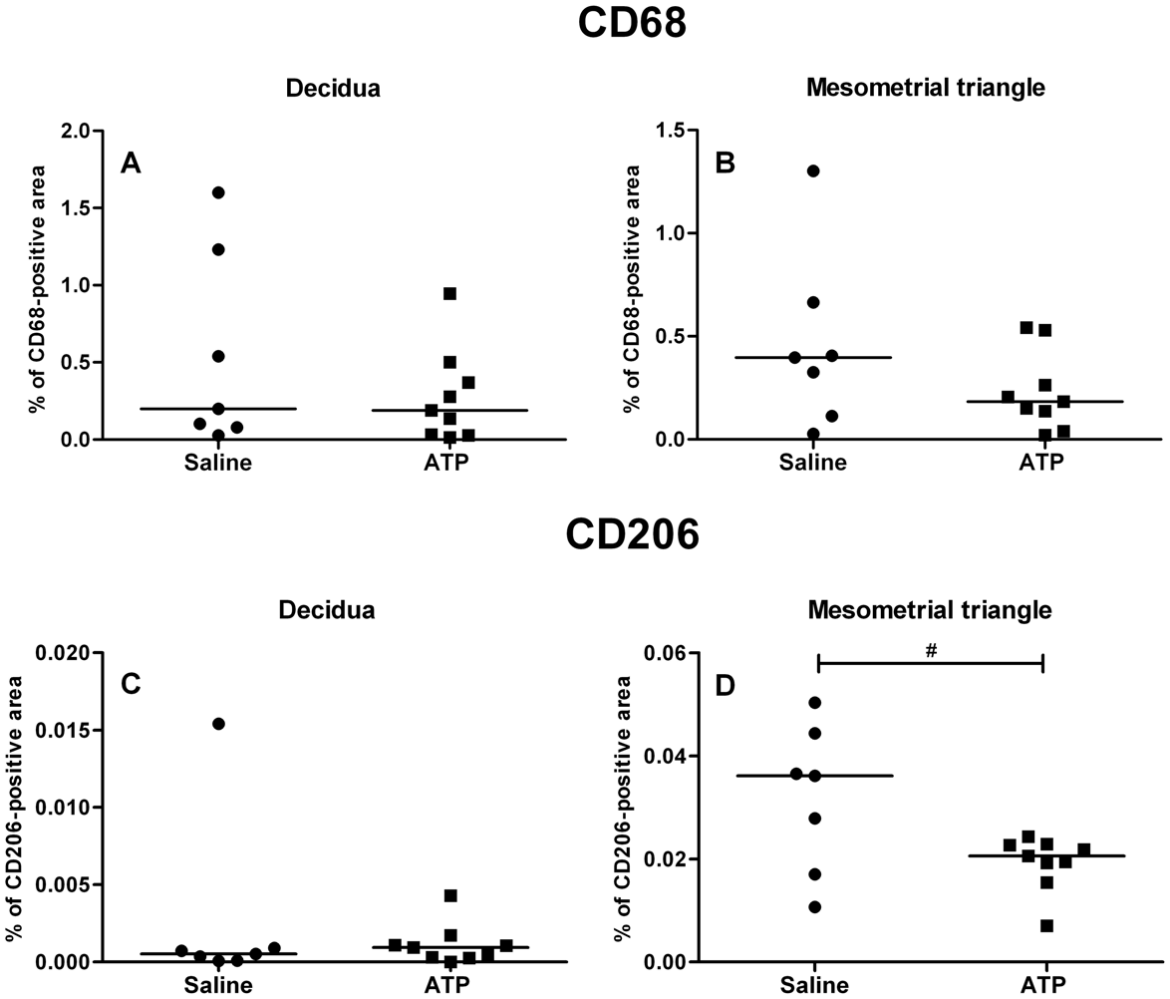
Total and CD206-positive macrophages in placentas of saline and ATP-infused pregnant rats. No changes in total, CD68-positive macrophages (as percentage positively stained tissue) were found after ATP-infusion (squares) in the decidua (A) and mesometrial triangle (B) as compared to saline-infusion (circles). Staining for CD206-positive macrophages was found to be lower in mesometrial tissue (D) of rats infused with ATP compared to saline. No differences were found for CD206 in decidual tissue between ATP and saline-infused rats (C). Medians are shown, #p<0.1; Mann Whitney U test.

Most of the staining for CD68-positive macrophages was located in the lower part of the decidua neighboring the trophospongium, while only few CD206-positive macrophages were found in the decidua. We found no differences in the percentage area stained for CD68-positive and CD206-positive decidual macrophages between saline and ATP-infused rats (Figures 7A and 7C).

Macrophages in the mesometrial triangle were located throughout the interstitium and around spiral arteries. We found no differences in the percentage area stained for CD68-positive mesometrial macrophages between saline and ATP-infused rats (Figure 7B). CD206-positive macrophages were mainly located around spiral arteries in the outer rim of the mesometrial triangle close to the longitudinal myometrial muscle layer. The percentage area stained for these macrophages was found to be lower (p<0.1, trend) in ATP-infused rats as compared to saline-infused rats (Figure 7D).

## Discussion

This study shows that the percentage of combined nonclassical/intermediate monocytes is higher during pregnancy in humans and in rats as compared to nonpregnant controls. This increase in nonclassical/intermediate monocytes is in accordance with the general view that pregnancy resembles a pro-inflammatory condition [8]. Most studies on monocyte subsets have focused on the combined nonclassical/intermediate monocytes. In the present communication we also investigated the separate intermediate and nonclassical subsets present in humans. We found that a higher percentage of intermediate monocytes in contrast to nonclassical monocytes is responsible for the higher percentage of combined nonclassical/intermediate monocytes. In pregnant women with preeclampsia, a condition with a further enhanced pro-inflammatory status as compared to healthy pregnancy [20], these differences were even more prominent. Moreover, data obtained in pregnant rats suggest that the higher percentage of nonclassical monocytes is induced by circulating pro-inflammatory stimuli present during preeclampsia (ATP in the present study) and is accompanied by lower numbers of regulatory/M2-like macrophages around the spiral arteries in the mesometrial triangle.

The changes in monocyte subsets observed in this study support the notion that pregnancy is a pro-inflammatory condition, which is even more prominent in preeclampsia. Immunologically, pregnancy is characterized by a shift from Th1 towards Th2 responses and a generalized activation of nonspecific immunity [8], [44], [45]. The observed changes in monocyte subsets in the third trimester of pregnancy may fit well within this altered inflammatory status, as higher numbers of combined nonclassical/intermediate monocytes have been associated with several inflammatory diseases [46]–[49]. For instance, higher numbers of circulating combined nonclassical/intermediate monocytes have been reported in sepsis, rheumatoid arthritis, HIV-1 infection, metastatic cancer, tuberculosis and asthma [46]–[49]. The combined nonclassical/intermediate monocyte subset consists of 2 subpopulations and our data indicate that an increase in intermediate monocytes is responsible for the higher percentage of this combined subset in healthy pregnancy and preeclampsia. Data from other inflammatory conditions (sepsis, asthma, rheumatoid arthritis, atherosclerosis, HIV-1 infection) confirm that intermediate monocytes are specifically found to be present in higher numbers during inflammation [46], [48], [50], [51].

Although the exact functions of the different monocyte subsets remain uncertain, compared to classical monocytes, combined nonclassical/intermediate monocytes produce higher amounts of TNF-α and IL-1ß after stimulation, exhibit lower phagocytic activity and have higher antigen presenting capacity [15], [46], [51]–[54]. With respect to the function of the various subsets of monocytes in pregnancy, no specific data have been published to our knowledge. In a nonpregnant situation, studies have shown that the transcriptome of the intermediate subset is consistent with pro-inflammatory characteristics and that these monocytes show high production of toxic oxygen radicals [51]. Data from other studies suggest that nonclassical monocytes, as opposed to intermediate monocytes, are prepared to move out of the circulation because they have the highest expression of genes involved in adhesion and transendothelial migration [51], [55], [56]. It would therefore appear that nonclassical monocytes have a role in tissues, because they show a phenotype that allows extravasation and that intermediate monocytes have a role as circulating, pro-inflammatory cells representing a first line of host defense. An increased activational state of these cells may be necessary to compensate for the adaptation of adaptive immunity during pregnancy, i.e. a shift away from a Th1 type immune responses [44].

As functional studies into human monocyte subsets *in vivo* are limited, we explored whether the changes we found in pregnant women correlated with pregnant rats. In rats, only two monocyte subsets are found. These are characterized by high expression of CD172a (signal-regulatory protein alpha) and either low or high expression of CD43 (leukosialin) [18]. CD172a+ CD43-lo monocytes have been found to be comparable to classical monocytes and CD172a+ CD43-hi monocytes to nonclassical monocytes, with no further subdivision reported of these nonclassical monocytes [14]. At the end of pregnancy, in healthy pregnant rats, we found higher percentages of total monocytes on average as compared to nonpregnant animals, similar to the human situation. Moreover, the nonclassical subset was also higher during rat pregnancy, similar to human pregnancy. This increase in nonclassical monocytes was already observed from day 13 of pregnancy until the end of pregnancy, suggesting that the pro-inflammatory condition in the rat develops in the second week of pregnancy. Whether this timing will be similar in humans remains to be investigated. However, previously it has been shown that phenotypical monocyte activation is increased in the second trimester and further increased in the third trimester [7], suggesting that also in human pregnancy the pro-inflammatory condition develops during the second trimester.

To investigate whether the higher percentage of nonclassical monocytes in preeclampsia could be induced by pro-inflammatory stimuli, we infused pregnant rats with ATP, a pro-inflammatory stimulus [29]. We found a further increase in nonclassical monocytes in pregnant rats infused with ATP as compared to control pregnant rats infused with saline. This supports our hypothesis that a pro-inflammatory stimulus in preeclampsia is responsible for the increase in nonclassical monocytes in this condition. In addition, in women with preeclampsia higher levels of ATP are observed as compared with healthy pregnant individuals, suggesting that in preeclampsia ATP may play a pathophysiological role [28].

The increase in the percentage of nonclassical monocytes after ATP infusion is accompanied by a higher activation status of this monocyte subset compared to saline treatment. To assess their activation status we used two markers known to be downregulated during activation of monocytes, i.e. CD62L (L-selectin) and CD4. CD62L is rapidly, but transiently, shed after pro-inflammatory signals [41], [42], while CD4 is down regulated for longer periods of time [40], [43]. The shedding of CD62L is thought to be a protective mechanism against excessive or aberrant monocyte recruitment [42], while the function of CD4 down regulation remains unknown. Interestingly, ATP does not have these effects on monocytes from nonpregnant animals or on classical monocytes from pregnant animals. Apparently the pregnant condition per sé alters the responsiveness of nonclassical monocytes leading to enhanced activation after a pro-inflammatory stimulus. We speculate that the characteristics of preeclampsia following infusion of ATP into pregnant rats are mediated (at least in part) by enhanced activity of nonclassical monocytes [29], [57].

Our study shows that changes in monocyte subsets in the peripheral circulation in our rat model appear to be accompanied by changes in macrophage subsets in the mesometrial triangle, i.e. the placental bed. A trend towards decreased numbers of M2–like or regulatory macrophages was observed in the mesometrial triangle on day 20 of pregnancy in ATP-infused rats as compared with saline-infused control rats. This may be similar to the human situation in which we have previously shown lower expression of M2 macrophage markers in deciduas of pregnancies later complicated with pregnancy-induced hypertension [58]. However, our studies still not answer the question how numbers and type of peripheral blood monocytes and placental macrophages are related. From mouse studies, it has been suggested that classical monocytes infiltrate acutely inflamed tissue [59], while nonclassical monocytes are suggested to replenish tissue macrophages in steady state conditions [27], [60]. It is tempting to speculate that during normal pregnancy nonclassical monocytes infiltrate the mesometrial triangle and that this process is inhibited after ATP infusion in pregnant rats.

The exact mechanism which induced the higher percentages of nonclassical monocytes in pregnancy and preeclampsia remains to be established. ATP, as suggested above, but also pro-inflammatory cytokines released from the placenta may be responsible [61], as well as shedding of syncytiotrophoblast membrane microparticles (STBMs) [62]. STBMs have been found to activate monocytes and are significantly increased during preeclampsia [63]–[65], but unfortunately none of these studies looked into the different monocyte subsets. Recent studies focusing on the interaction between activated platelets and monocytes may also offer an explanation for increased intermediate monocytes [66]. Preeclampsia is characterized by platelet activation [67] and these studies showed that activated platelets expand the pool of intermediate monocytes in a cyclooxygenase-2-dependent manner [66].

In conclusion, during pregnancy in humans and in rats the percentage of the pro-inflammatory combined nonclassical/intermediate monocytes is higher, which is in line with the pro-inflammatory status of healthy pregnancy. In humans this increase is caused by an increase in intermediate monocytes within this combined nonclassical/intermediate monocyte subset. During preeclampsia intermediate monocytes are even further increased. The observation that ATP enhanced numbers and activation of nonclassical monocytes in pregnant rats only, suggests that this subset is specifically altered in pregnancy and that they may play a role in the pathophysiology of preeclampsia. Whether this specific subsets infiltrates the mesometrial triangle, i.e. placental bed in healthy pregnancy, remains to be established.
